# Obstetric and Perinatal Outcomes After Assisted Reproductive Technology in Women With Cesarean Scar

**DOI:** 10.3389/fphys.2022.808079

**Published:** 2022-02-17

**Authors:** Yue Lin, Qianqian Chen, Xuefeng Huang, Ziliang Wang, Cuie Chen, Haiying Chen, Fan Jin

**Affiliations:** ^1^Department of Reproductive Endocrinology, Women’s Hospital, Zhejiang University School of Medicine, Hangzhou, China; ^2^Reproductive Medicine Center, First Affiliated Hospital of Wenzhou Medical University, Wenzhou, China; ^3^NHC Key Laboratory of Reproduction Regulation (Shanghai Institute for Biomedical and Pharmaceutical Technologies), School of Public Health, Fudan University, Shanghai, China; ^4^Department of Obstetrics and Gynecology, Affiliated Yueqing Hospital of Wenzhou Medical University, Wenzhou, China; ^5^Department of Obstetrics and Gynecology, Wenzhou Central Hospital, Wenzhou Maternal and Child Health Care Hospital, Wenzhou, China; ^6^Women’s Reproductive Health Laboratory of Zhejiang Province, Key Laboratory of Reproductive Genetics, National Ministry of Education (Zhejiang University), Hangzhou, China

**Keywords:** assisted reproductive techniques, Cesarean section, complications, interaction, offspring health, safety

## Abstract

**Introduction:**

Assisted reproductive technology (ART) and previous Cesarean section (CS) are independently associated with the risk of adverse obstetric and perinatal outcomes in general. Few studies have focused on the association between adverse obstetric and perinatal outcomes and ART used in the high-risk population of women with previous CS.

**Materials and Methods:**

A retrospective cohort study including 14,099 women with a previous delivery and a subsequent delivery between April 2014 and April 2020 was conducted at our hospital. We assessed the risk of adverse obstetric and perinatal outcomes in pregnancies conceived by ART in women with previous CS, using log-binomial regression models.

**Results:**

In women with previous CS, ART singleton pregnancies were associated with an increased risk of maternal complications, such as pregnancy complications, placental anomalies of implantation, postpartum hemorrhage, and preterm birth (PTB), as compared to spontaneously conceived pregnancies. The implementation of ART and previous CS interacted in a synergistic manner to increase the likelihood of the placenta accreta spectrum in women with singleton pregnancies [adjusted relative risk (aRR) 5.30, 95% confidence interval (CI) 4.01–7.00; relative risk due to interaction: 1.41, 95%CI 0.07–2.75]. In women with previous CS who underwent ART, women with singletons conceived through intracytoplasmic sperm injection were at increased risk of velamentous placenta (aRR 2.46, 95%CI 1.35–4.48) compared with those with singletons conceived through *in vitro* fertilization (IVF), whereas women with singletons conceived through cleavage-stage embryo transfer (ET) were at increased risk of gestational diabetes mellitus (GDM) (aRR 1.74, 95%CI 1.16-2.60) than those with singletons conceived through blastocyst–stage ET.

**Conclusion:**

Pregnancies conceived through ART were at increased risk for adverse obstetric and perinatal outcomes in women who had previously delivered by CS, particularly for placental anomalies of implantation. In women with previous CS undergoing ART, IVF and blastocyst–stage ET may be a relatively safe treatment.

## Introduction

It is well-documented that Cesarean section (CS) might increase the incidence of adverse obstetric and perinatal outcomes in subsequent conceptions, including persistent complete placenta previa, placental abruption, uterine Cesarean scar rupture, preterm birth (PTB), and low birth weight (LBW) (Ventura Laveriano and Redondo, 2013; Hu et al., 2018; Granfors et al., 2020). Thus, women with a Cesarean scar are a high–risk population for obstetric and perinatal complications in subsequent conceptions. Over the past decade, the number of infertile women with a Cesarean scar who seek assisted reproductive technology (ART) has been steadily increasing (Zhang et al., 2016). However, pregnancies conceived through ART have been suggested to have a higher risk of adverse obstetric and perinatal outcomes than spontaneously conceived (SC) pregnancies (Qin et al., 2016; Vannuccini et al., 2018; Yanaihara et al., 2018). Hence, the prevalence of adverse obstetric and perinatal outcomes in pregnancies conceived by ART in women with previous CS should be investigated. Nevertheless, few studies have focused on this topic. In addition, little is known about the effect of the type of ART procedure used in such women in relation to obstetric and perinatal outcomes.

The present retrospective cohort study aimed to assess the prevalence of adverse obstetric and perinatal outcomes associated with ART in women with previous CS precisely and to elucidate how to implement ART safely in infertile women with a Cesarean scar.

## Materials and Methods

### Study Design and Participants

We conducted a retrospective cohort study, including all multipara women with a single previous full-term delivery and a subsequent delivery between April 2014 and April 2020 at the First Affiliated Hospital of Wenzhou Medical University. Obstetric and perinatal data of live newborns delivered after the 28th week of gestation were obtained from the delivery records. The exclusion criteria were as follows: (1) congenital uterine malformations, including uterus unicornis, uterus bicornis, septate uterus, and duplex uterus; (2) previous uterine myomectomy; and (3) stillbirths at current delivery. ART included *in vitro* fertilization (IVF)/intracytoplasmic sperm injection (ICSI)–embryo transfer (ET), and frozen-thawed ET (FET). In women with a history of CS, pregnancies conceived through ART were assigned to the CS–ART group, whereas spontaneously conceived pregnancies were categorized into the CS–SC group. In women with a history of vaginal delivery (VD), pregnancies conceived through ART were assigned to the VD–ART group, whereas spontaneously conceived pregnancies were categorized into the VD–SC group.

Subsequently, the ART pregnancy group was divided into IVF and ICSI subgroups according to the fertilization mode, into the ET and FET subgroups according to different ET methods, and into blastocyst and cleavage-stage ET subgroups according to different embryo developmental stages. Using the unique personal identification number, all data were retrospectively collected from computer databases and stored in a deidentified database. Validation was performed on the data to check for errors and inconsistencies in documentation and coding.

### Outcomes

The study outcomes consisted of four parts: pregnancy complications, including gestational hypertension, preeclampsia, gestational diabetes mellitus (GDM); placental anomalies of implantation (Vahanian et al., 2015; Jauniaux et al., 2020), including placenta previa, low-lying placenta, velamentous placenta, placenta accreta spectrum (defined as abnormal adherence of the placenta to the implantation site) (Miller et al., 2021); other complications, including placental abruption, postpartum hemorrhage, uterine rupture, preterm prelabor rupture of the membranes (pPROM, defined as rupture of the fetal membranes prior to 37 weeks of completed gestation), and perinatal outcomes, including PTB (delivery at < 37 completed weeks of gestation), very PTB (gestational age < 32 weeks), LBW (weight < 2,500 g), macrosomia (weight > 4,000 g), and Apgar score < 7 at 1 min.

### Statistical Analysis

Categorical variables are presented as numbers with percentages. In women with previous CS, the risks of obstetric and perinatal outcomes in ART pregnancies (vs. non-ART) stratified by birth plurality were assessed using log-binomial regression models. The adjusted risk ratios (aRRs) with 95% confidence intervals (CIs) for each outcome and the interaction models were calculated after controlling for maternal age at the time of delivery (≤ 35, 36–39, and ≥ 40 years), interpregnancy interval (< 6, 6–12, and > 12 months) (Kangatharan et al., 2017), other previous intrauterine operation, body mass index at the time of delivery (< 24 and ≥ 24 kg/m^2^), and education level (≤ 9 and > 9 years). Other previous intrauterine operations included curettage, surgical termination of pregnancy, and evacuation of retained conception products. In the ART pregnancy group of women with previous CS, we compared the incidence of obstetric and perinatal complications between different fertilization modes, between different ET methods, and between different embryo developmental stages, to elucidate how to implement ART safely in infertile women with a Cesarean scar. For comparison between different fertilization modes, we additionally adjusted for ET methods and embryo developmental stages. For comparison between different ET methods, we additionally adjusted for fertilization modes and embryo developmental stages. For comparison between different embryo developmental stages, we additionally adjusted for fertilization modes and ET methods. For maternal outcomes, such as pregnancy complications, placental anomalies of implantation and other obstetric complications, as the dependent variable, the unit of analysis was the delivery. For perinatal outcomes as the dependent variable, the unit of analysis was the offspring. In addition, for perinatal outcomes in twin pregnancies, generalized estimating equations were used to account for the correlation between the twins of the same mother (Grove et al., 1993).

In addition, we investigated the interaction between ART implementation and previous CS on the risk of obstetric and perinatal complications stratified by birth plurality, in which the VD–SC group were used as the reference group. The interaction measure between ART implementation and previous CS on the risk of obstetric and perinatal complications should only be used if the two exposure factors are risk factors for obstetric and perinatal outcomes. The relative excess risk due to interaction (RERI) along with 95%CI on the additive scale were calculated using the method described by Hosmer and Lemeshow (1992). RERI represents the extent to which risk increases due to the interaction of two exposures, rather than the sum of the individual risks (Knol and VanderWeele, 2012). Positive interactions on the additive scale were represented by a RERI greater than 0. A positive interaction on the additive scale indicates that the estimated joint effect of the two exposure factors exceeds the sum of their individual effects. SPSS statistical software (version 25; SPSS Inc., Armonk, NY, United States) was used for data analysis.

### Ethics Approval

All participants provided informed consent before undergoing routine treatment. Using the unique personal identification number, all data were retrospectively collected from computer databases and stored in a deidentified database. This study was approved by the ethics committee of the First Affiliated Hospital of Wenzhou Medical University (no. 2020–04) and performed according to the principles embodied in the Declaration of Helsinki.

## Results

**FIGURE 1:**
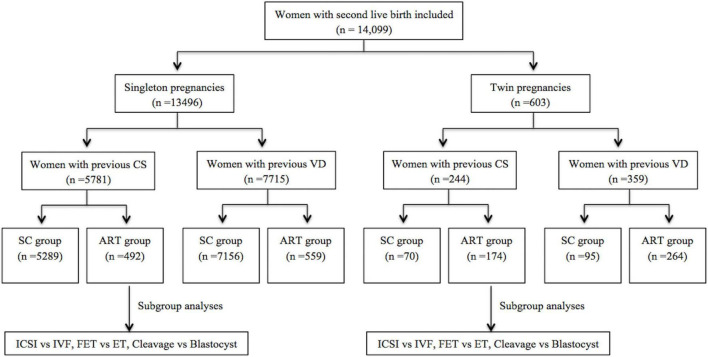
Flow chart of the population in a study of obstetric and perinatal outcomes in women with a single previous full-term delivery. ART, assisted reproductive technology; SC, spontaneously conceived; CS, Cesarean section; VD, vaginal delivery; IVF, *in vitro* fertilization; ICSI, intracytoplasmic sperm injection; ET, embryo transfer; FET, frozen-thawed ET.

A total of 14,099 participants were included in the analysis (Figure 1). We identified 6,025 women with previous CS and 8,074 women with previous VD. Of the women with previous CS, 666 were in the CS-ART group and 5,359 were in the CS–SC group. Of the women with previous VD, 823 were in the VD-ART group and 7,251 were in the VD–SC group.

The maternal characteristics of singleton pregnancies and twin pregnancies are shown in Tables 1, 2, respectively. Obstetric and perinatal outcomes of singleton pregnancies are summarized in Table 3, and those of twin pregnancies are shown in Table 4. Uterine rupture occurred in 18 women with previous CS, 14 of whom were in CS–SC group, whereas none of the women with previous VD had uterine rupture. An overwhelming majority (> 90%) of women with previous CS had subsequent CS delivery, regardless of conception method and birth plurality.

**TABLE 1:** Maternal characteristics in subsequent singleton pregnancies according to the mode of delivery at the first birth.

	Previous CS								Previous VD
	Spontaneous conception (*n* = 5,289)	ART (*n* = 492)	IVF (*n* = 365)	ICSI (*n* = 127)	Fresh ET (*n* = 173)	FET (*n* = 319)	Cleavage (*n* = 360)	Blastocyst (*n* = 132)	Spontaneous conception (*n* = 7,156)

	*n* (%)	*n* (%)	*n* (%)	*n* (%)	*n* (%)	*n* (%)	*n* (%)	*n* (%)	*n* (%)
**Maternal age**									
≤ 35 years	4,106 (77.6)	276 (56.1)	196 (53.7)	80 (63.0)	98 (56.6)	178 (55.8)	186 (51.7)	90 (68.2)	5,823 (81.4)
36–39 years	934 (17.7)	150 (30.5)	115 (31.5)	35 (27.6)	55 (31.8)	95 (29.8)	118 (32.8)	32 (24.2)	978 (13.7)
≥ 40 years	249 (4.7)	66 (13.4)	54 (14.8)	12 (9.4)	20 (11.6)	46 (14.4)	56 (15.6)	10 (7.6)	355 (5.0)
**Cause of infertility**									
PCOS	ND^a^	54 (11.0)	42 (11.5)	12 (9.4)	0 (0.0)	54 (16.9)	33 (9.2)	21 (15.9)	ND^a^
Tubal factor	ND^a^	343 (69.7)	259 (71.0)	84 (66.1)	109 (63.0)	234 (73.4)	241 (66.9)	102 (77.3)	ND^a^
Endometriosis	ND^a^	44 (8.9)	36 (9.9)	8 (6.3)	9 (5.2)	35 (11.0)	26 (7.2)	18 (13.6)	ND^a^
Male factor	ND^a^	188 (38.2)	72 (19.7)	116 (91.3)	57 (32.9)	131 (41.1)	139 (38.6)	49 (37.1)	ND^a^
Unexplained	ND^a^	56 (11.4)	45 (12.3)	11 (8.7)	19 (11.0)	37 (11.6)	37 (10.3)	19 (14.4)	ND^a^
**Interpregnancy interval**									
< 6 months	158 (3.0)	24 (4.9)	24 (6.6)	0 (0.0)	12 (6.9)	12 (3.8)	16 (4.4)	8 (6.1)	247 (3.5)
6–12 months	322 (6.1)	92 (18.7)	73 (20.0)	19 (15.0)	28 (16.2)	64 (20.1)	77 (21.4)	15 (11.4)	4,445 (62.1)
> 12 months	4,809 (90.9)	376 (76.4)	268 (73.4)	108 (85.0)	133 (76.9)	243 (76.2)	267 (74.2)	109 (82.6)	2,464 (34.4)
**First birth conceived through ART**									
Yes	0 (0.0)	126 (25.6)	84 (23.0)	42 (33.1)	46 (26.6)	80 (25.1)	82 (22.8)	44 (33.3)	0 (0.0)
No	5,289 (100.0)	366 (74.4)	281 (77.0)	85 (66.9)	127 (73.4)	239 (74.9)	278 (77.2)	88 (66.7)	7,156 (100.0)
**Education level**									
≤ 9 years	3,188 (60.3)	293 (59.6)	219 (60.0)	74 (58.3)	95 (54.9)	198 (62.1)	206 (57.2)	87 (65.9)	4,634 (64.8)
> 9 years	2,101 (39.7)	199 (40.4)	146 (40.0)	53 (41.2)	78 (45.1)	121 (37.9)	154 (42.8)	45 (34.1)	2,522 (35.2)
**Smoking**									
Yes	0 (0.0)	2 (0.4)	2 (0.5)	0 (0.0)	0 (0.0)	2 (0.6)	1 (0.3)	1 (0.8)	0 (0.0)
No	5,289 (100.0)	490 (99.6)	363 (99.5)	127 (100.0)	173 (100.0)	317 (99.4)	359 (99.7)	131 (99.2)	7,156 (100.0)
**Alcohol consumption**									
Yes	1 (0.0)	0 (0.0)	0 (0.0)	0 (0.0)	0 (0.0)	0 (0.0)	0 (0.0)	0 (0.0)	3 (0.0)
No	5,288 (100.0)	492 (100.0)	365 (100.0)	127 (100.0)	173 (100.0)	319 (100.0)	360 (100.0)	132 (100.0)	7,153 (100.0)
**Other previous intrauterine operation** ^b^									
Yes	4,637 (87.7)	444 (90.2)	322 (88.2)	122 (96.1)	160 (92.5)	284 (89.0)	327 (90.8)	117 (88.6)	6,381 (89.2)
No	652 (12.3)	48 (9.8)	43 (11.8)	5 (3.9)	13 (7.5)	35 (11.0)	33 (9.2)	15 (11.4)	775 (10.8)
**Maternal body mass index**									
< 24 kg/m^2^	777 (14.7)	147 (29.9)	106 (29.0)	41 (32.3)	42 (24.3)	105 (32.9)	97 (26.9)	50 (37.9)	1,516 (21.2)
≥ 24 kg/m^2^	4,512 (85.3)	345 (70.1)	259 (71.0)	86 (67.7)	131 (75.7)	214 (67.1)	263 (73.1)	82 (62.1)	5,640 (78.8)

*ART, assisted reproductive technology; CS, Cesarean section; VD, vaginal delivery; IVF, in vitro fertilization; ICSI, intracytoplasmic sperm injection; ET, embryo transfer; FET, frozen-thawed ET; ND, not defined. Cause of infertility of someone may be more than 1 cause possible.*

*^a^Because of zero counts in one cell.*

*^b^Included curettage, surgical termination of pregnancy and evacuation of retained products of conception.*

**TABLE 2:** Maternal characteristics in subsequent twin pregnancies according to mode of delivery at the first birth.

	Previous CS									Previous VD
	Spontaneous conception (*n* = 70)	ART (*n* = 174)	IVF (*n* = 126)	ICSI (*n* = 48)	Fresh ET (*n* = 69)	FET (*n* = 105)		Cleavage (*n* = 132)	Blastocyst (*n* = 42)	Spontaneous conception (*n* = 95)

	*n* (%)	*n* (%)	*n* (%)	*n* (%)	*n* (%)	*n*	(%)	*n* (%)	*n* (%)	*n* (%)
**Maternal age**										
≤ 35 years	56 (80.0)	116 (66.7)	83 (65.9)	33 (68.8)	48 (69.6)	68 (64.8)		83 (62.9)	33 (78.6)	83 (87.4)
36–39 years	10 (14.3)	51 (29.3)	37 (29.4)	14 (29.3)	18 (26.1)	33 (31.4)		43 (32.6)	8 (19.0)	10 (10.5)
≥ 40 years	4 (5.7)	7 (4.0)	6 (4.8)	1 (4.0)	3 (4.3)	4 (3.8)		6 (4.5)	1 (2.4)	2 (2.1)
**Cause of infertility**										
PCOS	ND^a^	22 (12.6)	16 (12.7)	6 (12.5)	0 (0.0)	22 (21.0)		16 (12.1)	6 (14.3)	ND^a^
Tubal factor	ND^a^	115 (66.1)	104 (82.5)	11 (22.9)	47 (68.1)	68 (64.8)		88 (66.7)	27 (64.3)	ND^a^
Endometriosis	ND^a^	27 (15.5)	20 (15.9)	7 (14.6)	6 (8.7)	21 (20.0)		21 (15.9)	6 (14.3)	ND^a^
Male factor	ND^a^	82 (47.1)	41 (32.5)	41 (85.4)	28 (40.6)	54 (51.4)		51 (38.6)	31 (73.8)	ND^a^
Unexplained	ND^a^	6 (3.4)	6 (4.8)	0 (0.0)	3 (4.3)	3 (2.9)		6 (4.5)	0 (0.0)	ND^a^
**Interpregnancy interval**										
< 6 months	3 (4.3)	4 (2.3)	2 (1.6)	2 (4.2)	0 (0.0)	4 (3.8)		2 (1.5)	2 (4.8)	5 (5.3)
6–12 months	9 (12.9)	40 (23.0)	27 (21.4)	13 (27.1)	14 (20.3)	26 (24.8)		34 (25.8)	6 (14.3)	76 (80.0)
> 12 months	58 (82.9)	130 (74.7)	97 (77.0)	33 (68.8)	55 (79.7)	75 (71.4)		96 (72.7)	34 (81.0)	14 (14.7)
**First birth conceived through ART**										
Yes	0 (0.0)	45 (25.9)	28 (22.2)	17 (35.4)	12 (17.4)	33 (31.4)		28 (21.2)	17 (40.5)	0 (0.0)
No	70 (100.0)	129 (74.1)	98 (77.8)	31 (64.6)	57 (82.6)	72 (68.6)		104 (78.8)	25 (59.5)	95 (100.0)
**Education level**										
≤ 9 years	52 (74.3)	96 (55.2)	70 (55.6)	26 (54.2)	48 (69.6)	48 (45.7)		71 (53.8)	25 (59.5)	81 (85.3)
> 9 years	18 (25.7)	78 (44.8)	56 (44.4)	22 (45.8)	21 (30.4)	57 (54.3)		61 (46.2)	17 (40.5)	14 (14.7)
**Smoking**										
Yes	0 (0.0)	1 (0.6)	1 (0.8)	0 (0.0)	0 (0.0)	1 (1.0)		1 (0.8)	0 (0.0)	0 (0.0)
No	70 (100.0)	173 (99.4)	125 (99.2)	48 (100.0)	69 (100.0)	104 (99.0)		131 (99.2)	42 (100.0)	95 (100.0)
**Alcohol consumption**										
Yes	0 (0.0)	0 (0.0)	0 (0.0)	0 (0.0)	0 (0.0)	0 (0.0)		0 (0.0)	0 (0.0)	0 (0.0)
No	70 (100.0)	174 (100.0)	126 (100.0)	48 (100.0)	69 (100.0)	105 (100.0)		132 (100.0)	42 (100.0)	95 (100.0)
**Other previous intrauterine operation** ^b^										
Yes	58 (82.9)	162 (93.1)	116 (92.1)	46 (95.8)	61 (88.4)	101 (96.2)		123 (93.2)	39 (92.9)	89 (90.5)
No	12 (17.1)	12 (6.9)	10 (7.9)	2 (4.2)	8 (11.6)	4 (3.8)		9 (6.8)	3 (7.1)	9 (9.5)
**Maternal body mass index**										
< 24 kg/m^2^	6 (82.9)	57 (32.8)	41 (32.5)	16 (33.3)	22 (31.9)	35 (33.3)		42 (31.8)	15 (35.7)	7 (7.4)
≥ 24 kg/m^2^	64 (91.4)	117 (67.2)	85 (67.5)	32 (66.7)	47 (68.7)	70 (66.7)		90 (68.2)	27 (64.3)	88 (92.6)

*ART, assisted reproductive technology; CS, Cesarean section; VD, vaginal delivery; IVF, in vitro fertilization; ICSI, intracytoplasmic sperm injection; ET, embryo transfer; FET, frozen-thawed ET; ND, not defined. Cause of infertility of someone may be more than 1 cause possible.*

*^a^Because of zero counts in one cell.*

*^b^Included curettage, surgical termination of pregnancy and evacuation of retained products of conception.*

**TABLE 3:** Obstetric and perinatal outcomes in subsequent singleton pregnancies according to mode of delivery at the first birth.

	Previous CS								Previous VD
	Spontaneous conception (*n* = 5,289)	ART (*n* = 492)	IVF (*n* = 365)	ICSI (*n* = 127)	Fresh ET (*n* = 173)	FET (*n* = 319)	Cleavage (*n* = 360)	Blastocyst (*n* = 132)	Spontaneous conception (*n* = 7,156)

	*n* (%)	*n* (%)	*n* (%)	*n* (%)	*n* (%)	*n* (%)	*n* (%)	*n* (%)	*n* (%)
**Pregnancy complications**									
Gestational hypertension	169 (3.2)	43 (8.7)	30 (8.2)	13 (10.2)	9 (5.2)	34 (10.7)	25 (6.9)	18 (13.6)	195 (2.7)
Preeclampsia	110 (2.1)	16 (3.3)	10 (2.7)	6 (4.7)	6 (3.5)	10 (3.1)	9 (2.5)	7 (5.3)	109 (1.5)
GDM	907 (17.1)	131 (26.6)	91 (24.9)	40 (31.5)	47 (27.2)	84 (26.3)	109 (30.3)	22 (16.7)	937 (13.1)
**Placental anomalies of implantation**									
Placenta previa	132 (2.5)	19 (3.9)	18 (4.9)	1 (0.8)	6 (3.5)	13 (4.1)	15 (4.2)	4 (3.0)	102 (1.4)
Low-lying placenta	33 (0.6)	7 (1.4)	5 (1.4)	2 (1.6)	1 (0.6)	6 (1.9)	6 (1.7)	1 (0.8)	41 (0.6)
Velamentous placenta	186 (3.5)	42 (8.5)	26 (7.1)	16 (12.6)	10 (5.8)	32 (10.0)	29 (8.1)	13 (9.8)	354 (4.9)
Placenta accreta spectrum	409 (7.7)	85 (17.3)	68 (18.6)	17 (13.4)	21 (12.1)	64 (20.1)	60 (16.7)	25 (18.9)	203 (2.8)
**Other complications**									
Placental abruption	38 (0.7)	5 (1.0)	3 (0.8)	2 (1.6)	0 (0.0)	5 (1.6)	4 (1.1)	1 (0.8)	77 (1.1)
Postpartum hemorrhage	18 (0.3)	10 (2.0)	7 (1.9)	3 (2.4)	3 (1.7)	7 (2.2)	5 (1.4)	5 (3.8)	33 (0.5)
pPROM	544 (10.3)	55 (11.2)	47 (12.9)	8 (6.3)	21 (12.1)	34 (10.7)	48 (13.3)	7 (5.3)	1,403 (19.6)
Uterine rupture	14 (0.3)	3 (0.6)	3 (0.8)	0 (0.0)	1 (0.6)	2 (0.6)	3 (0.8)	0 (0.0)	0 (0.0)
Cesarean section	4,767 (90.1)	471 (95.7)	349 (95.6)	122 (96.1)	169 (97.7)	302 (94.7)	350 (97.2)	121 (91.7)	920 (12.9)
**Infants**									
PTB	394 (7.4)	55 (11.2)	46 (12.6)	9 (7.1)	24 (13.9)	31 (9.7)	48 (13.3)	7 (5.3)	482 (6.7)
Very PTB	51 (1.0)	9 (1.8)	9 (2.5)	0 (0.0)	3 (1.7)	6 (1.9)	7 (1.9)	2 (1.5)	73 (1.0)
LBW	181 (3.4)	26 (5.3)	23 (6.3)	3 (2.4)	11 (6.4)	15 (4.7)	22 (6.1)	4 (3.0)	212 (3.0)
Macrosomia	310 (5.9)	29 (5.9)	19 (5.2)	10 (7.9)	2 (1.2)	27 (8.5)	14 (3.9)	15 (11.4)	441 (6.2)
Apgar score < 7 at 1 min	67 (1.3)	6 (1.2)	6 (1.6)	0 (0.0)	4 (2.3)	2 (0.6)	3 (0.8)	3 (2.3)	73 (1.0)

*ART, assisted reproductive technology; CS, Cesarean section; VD, vaginal delivery; IVF, in vitro fertilization; ICSI, intracytoplasmic sperm injection; ET, embryo transfer; FET, frozen-thawed ET; GDM, gestational diabetes mellitus; pPROM, preterm prelabor rupture of the membranes; PTB, preterm birth; LBW, low birthweight.*

**TABLE 4:** Obstetric and perinatal outcomes in subsequent twin pregnancies according to mode of delivery at the first birth.

	Previous CS								Previous VD
	Spontaneous conception (*n* = 70)	ART (*n* = 174)	IVF (*n* = 126)	ICSI (*n* = 48)	Fresh ET (*n* = 69)	FET (*n* = 105)	Cleavage (*n* = 132)	Blastocyst (*n* = 42)	Spontaneous conception (*n* = 95)

	*n* (%)	*n* (%)	*n* (%)	*n* (%)	*n* (%)	*n* (%)	*n* (%)	*n* (%)	*n* (%)
**Pregnancy complications**									
Gestational hypertension	10 (14.3)	32 (18.4)	26 (20.6)	6 (12.5)	7 (10.1)	25 (23.8)	20 (15.2)	12 (28.6)	8 (8.4)
Preeclampsia	7 (10.0)	14 (8.0)	10 (7.9)	4 (8.3)	3 (4.3)	11 (10.5)	9 (6.8)	5 (11.9)	4 (4.2)
GDM	14 (20.0)	48 (27.6)	34 (27.0)	14 (29.2)	19 (27.5)	29 (27.6)	32 (24.2)	16 (38.1)	17 (17.9)
**Placental anomalies of implantation**									
Placenta previa	0 (0.0)	16 (9.2)	9 (7.1)	7 (14.6)	8 (11.6)	8 (7.6)	14 (10.6)	2 (4.8)	1 (1.1)
Low-lying placenta	0 (0.0)	5 (2.9)	4 (3.2)	1 (2.1)	3 (4.3)	2 (1.9)	4 (3.0)	1 (2.4)	1 (1.1)
Velamentous placenta	6 (8.6)	23 (13.2)	17 (13.5)	6 (12.5)	12 (17.4)	11 (10.5)	23 (17.4)	0 (0.0)	5 (5.3)
Placenta accreta spectrum	9 (12.9)	22 (12.6)	15 (11.9)	7 (14.6)	2 (2.9)	20 (19.0)	19 (14.4)	3 (7.1)	12 (12.6)
**Other complications**									
Placental abruption	1 (1.4)	1 (0.6)	0 (0.0)	1 (2.1)	0 (0.0)	1 (1.0)	1 (0.8)	0 (0.0)	0 (0.0)
Postpartum hemorrhage	0 (0.0)	10 (5.7)	7 (5.6)	3 (6.3)	4 (5.8)	6 (5.7)	8 (6.1)	2 (4.8)	0 (0.0)
pPROM	7 (10.0)	36 (20.7)	26 (20.6)	10 (20.8)	10 (14.5)	26 (24.8)	25 (18.9)	11 (26.2)	16 (16.8)
Uterine rupture	1 (1.4)	0 (0.0)	0 (0.0)	0 (0.0)	0 (0.0)	0 (0.0)	0 (0.0)	0 (0.0)	0 (0.0)
Cesarean section	69 (98.6)	167 (96.0)	119 (94.4)	48 (100.0)	64 (92.8)	103 (98.1)	127 (96.2)	40 (95.2)	82 (86.3)
**Infants**									
PTB	82/140 (58.6)	240/348 (69.0)	168/252 (66.7)	72/96 (75.0)	90/138 (65.2)	150/210 (71.4)	178/264 (67.4)	62/84 (73.8)	98/190 (51.6)
Very PTB	4/140 (2.9)	24/348 (6.9)	18/252 (7.1)	6/96 (6.3)	12/138 (8.7)	12/210 (5.7)	20/264 (7.6)	4/84 (4.8)	4/190 (2.1)
LBW	27/140 (19.3)	149/348 (42.8)	100/252 (39.7)	49/96 (51.0)	62/138 (44.9)	87/210 (41.4)	115/264 (43.6)	34/84 (40.5)	28/190 (14.7)
Macrosomia	0 (0.0)	0 (0.0)	0 (0.0)	0 (0.0)	0 (0.0)	0 (0.0)	0 (0.0)	0 (0.0)	0 (0.0)
Apgar score < 7 at 1 min	3/140 (2.1)	25/348 (7.2)	7/252 (2.8)	18/96 (18.8)	10/138 (7.2)	15/210 (7.1)	14/264 (5.3)	11/84 (13.1)	8/190 (4.2)

*ART, assisted reproductive technology; CS, Cesarean section; VD, vaginal delivery; IVF, in vitro fertilization; ICSI, intracytoplasmic sperm injection; ET, embryo transfer; FET, frozen-thawed ET; GDM, gestational diabetes mellitus; pPROM, preterm prelabor rupture of the membranes; PTB, preterm birth; LBW, low birthweight.*

### Comparison of Obstetric and Perinatal Outcomes in Women With Previous Cesarean Section Who Conceived by Assisted Reproductive Technology or Spontaneously, Stratified by Birth Plurality

In women with previous CS, ART singleton pregnancies were associated with an increased risk of gestational hypertension (aRR 2.06, 95%CI 1.27–3.35), GDM (aRR 1.39, 95%CI 1.15–1.67), velamentous placenta (aRR 2.46, 95%CI 1.70–3.56), placenta accreta spectrum (aRR 2.07, 95%CI 1.61–2.66), postpartum hemorrhage (aRR 8.65, 95%CI 3.83–19.57), and PTB (aRR 1.34, 95%CI 1.01–1.77), as compared to singletons in the CS–SC group (Table 5).

**TABLE 5:** The effect of ART procedures on obstetric and perinatal outcomes of singletons in women with previous CS.

	ART vs. Spontaneous conception		ICSI vs. IVF		FET vs. ET		Cleavage vs. Blastocyst	
	aRR^b^ (95%CI)	*P*-value	aRR^c^ (95%CI)	*P*-value	aRR^d^ (95%CI)	*P*-value	aRR^e^ (95%CI)	*P*-value
**Pregnancy complications**								
Gestational hypertension	2.06 (1.27–3.35)	0.003	1.39 (0.77–2.52)	0.280	1.38 (0.64–2.97)	0.411	0.53 (0.28–0.99)	0.047
Preeclampsia	1.51 (0.76–3.00)	0.243	1.83 (0.68–4.91)	0.230	0.50 (0.16–1.57)	0.231	0.27 (0.09-0.84)	0.024
GDM	1.39 (1.15–1.67)	<0.001	1.26 (0.93–1.73)	0.142	0.97 (0.71–1.32)	0.969	1.74 (1.16–2.60)	0.007
**Placental anomalies of implantation**								
Placenta previa	1.31 (0.75–2.28)	0.338	0.16 (0.02–1.20)	0.074	1.22 (0.44–3.35)	0.703	1.51 (0.47–4.83)	0.485
Low-lying placenta	1.69 (0.54–5.35)	0.370	0.92 (0.18–4.64)	0.914	5.08 (0.60–42.86)	0.136	4.21 (0.49–36.10)	0.190
Velamentous placenta	2.46 (1.70–3.56)	<0.001	2.46 (1.35–4.48)	0.003	1.75 (0.86–3.56)	0.122	0.85 (0.45–1.62)	0.625
Placenta accreta spectrum	2.07 (1.61–2.66)	<0.001	0.77 (0.47–1.27)	0.312	1.62 (1.00–2.63)	0.053	1.19 (0.75–1.87)	0.465
**Other complications**								
Placental abruption	1.82 (0.68–4.82)	0.231	1.39 (0.23–8.48)	0.719	ND^a^		2.52 (0.28–22.37)	0.407
Postpartum hemorrhage	8.65 (3.83–19.57)	<0.001	1.13 (0.30–4.32)	0.858	0.81 (0.17–3.80)	0.792	0.34 (0.08–1.53)	0.165
pPROM	0.93 (0.67–1.28)	0.646	0.49 (0.24–1.00)	0.049	1.10 (0.65–1.85)	0.719	2.69 (1.21–5.95)	0.015
Uterine rupture	2.66 (0.75–9.51)	0.132	ND^a^		1.60 (0.16–16.10)	0.690	ND^a^	
**Infants**								
PTB	1.34 (1.01–1.77)	0.045	0.52 (0.26–1.03)	0.059	0.87 (0.52–1.46)	0.603	2.24 (1.00–5.01)	0.050
Very PTB	1.21 (0.53–2.77)	0.650	ND^a^		0.89 (0.21–3.83)	0.871	1.05 (0.19–5.65)	0.959
LBW	1.42 (0.93–2.16)	0.097	0.34 (0.10–1.10)	0.070	0.87 (0.40–1.90)	0.719	1.67 (0.56–4.98)	0.362
Macrosomia	1.12 (0.76–1.65)	0.561	1.58 (0.76–3.30)	0.224	6.07 (1.39–26.59)	0.017	0.59 (0.28–1.26)	0.174
Apgar score < 7 at 1 min	0.98 (0.40–2.38)	0.966	ND^a^		0.11 (0.02–0.76)	0.025	0.13 (0.02–0.77)	0.025

*ART, assisted reproductive technology; CS, Cesarean section; VD, vaginal delivery; aRR, adjusted risk ratio; CI, confidence interval; ND, not defined; GDM, gestational diabetes mellitus; pPROM, preterm prelabor rupture of the membranes; PTB, preterm birth; LBW, low birthweight.*

*^a^Because of zero counts in one cell.*

*^b^RRs were adjusted for maternal age and body mass index at the time of delivery, interpregnancy interval, other previous intrauterine operation, and education level.*

*^c^RRs were adjusted for maternal age and body mass index at the time of delivery, interpregnancy interval, other previous intrauterine operation, education level, embryo transfer methods, and embryo developmental stage.*

*^d^RRs were adjusted for maternal age and body mass index at the time of delivery, interpregnancy interval, other previous intrauterine operation, education level, fertilization modes, and embryo developmental stage.*

*^e^RRs were adjusted for maternal age and body mass index at the time of delivery, interpregnancy interval, other previous intrauterine operation, education level, fertilization modes, and embryo transfer methods.*

**FIGURE 2:**
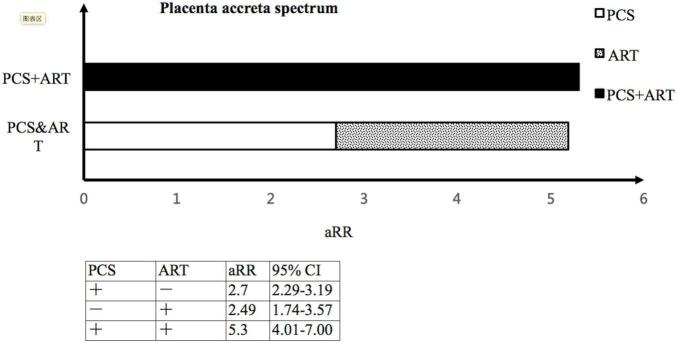
The interaction between the implementation of ART and previous CS on the risk of placenta accreta spectrum. ART, assisted reproductive technology; CS, Cesarean section; aRR, adjusted risk ratio; CI, confidence interval.

The implementation of ART and previous CS (Supplementary Table 1) are both risk factors for GDM and placenta accreta spectrum, when using VD–SC group as the reference group. We then investigated the interaction between the implementation of ART and previous CS on the risk of GDM and placenta accreta spectrum in singleton pregnancies (Supplementary Table 2). In singleton pregnancies, women with previous CS undergoing ART were found to have a significantly increased risk of placenta accreta spectrum (aRR 5.30, 95%CI 4.01-7.00; RERI 1.41, 95%CI 0.07–2.75), as compared to VD–SC group. This was due to a positive interaction on the additive scale between the implementation of ART and previous CS (Figure 2).

In women with previous CS, twins born following ART had an increased risk of LBW (aRR 2.34, 95% CI 1.37–3.98) compared to twins in CS–SC group (Table 6). Previous CS is not a risk factor for LBW in twin pregnancies (Supplementary Table 3); therefore, the interaction between ART implementation and previous CS on the risk of LBW in twin pregnancies was not assessed.

**TABLE 6:** The effect of ART procedures on obstetric and perinatal outcomes of twins in women with previous CS.

	ART vs. Spontaneous conception		ICSI vs. IVF		FET vs. ET		Cleavage vs. Blastocyst	
	aRR^b^ (95%CI)	*P*-value	aRR^c^ (95%CI)	*P*-value	aRR^d^ (95%CI)	*P*-value	aRR^e^ (95%CI)	*P*-value
**Pregnancy complications**								
Gestational hypertension	1.45 (0.70–3.01)	0.318	0.54 (0.24–1.22)	0.137	1.75 (0.71–4.35)	0.226	0.54 (0.26–1.11)	0.090
Preeclampsia	0.97 (0.38–2.51)	0.952	0.84 (0.28–2.51)	0.758	0.87 (0.22–3.51)	0.848	0.44 (0.15-1.32)	0.141
GDM	1.46 (0.75–2.86)	0.268	1.07 (0.63–1.79)	0.813	0.75 (0.41–1.40)	0.367	0.54 (0.29–1.01)	0.053
**Placental anomalies of implantation**								
Placenta previa	ND^a^		1.69 (0.67–4.31)	0.268	0.61 (0.23–1.59)	0.310	1.62 (0.34–7.87)	0.547
Low-lying placenta	ND^a^		0.62 (0.08–4.92)	0.647	0.07 (0.04-1.22)	0.068	0.46 (0.03–6.00)	0.549
Velamentous placenta	2.44 (0.96–6.19)	0.061	1.02 (0.44–2.39)	0.963	1.05 (0.48–2.31)	0.907	ND^a^	
Placenta accreta spectrum	1.44 (0.63–3.30)	0.395	1.48 (0.66–3.27)	0.340	7.28 (1.74–30.50)	0.007	3.38 (1.05–10.83)	0.041
**Other complications**								
Placental abruption	0.40 (0.03–6.34)	0.518	ND^a^		ND^a^		ND^a^	
Postpartum hemorrhage	ND^a^		1.22 (0.33–4.58)	0.766	1.05 (0.27–4.00)	0.949	1.24 (0.23–6.60)	0.799
pPROM	2.24 (0.97–5.18)	0.059	0.88 (0.46–1.68)	0.697	1.57 (0.75–3.30)	0.235	0.75 (0.39–1.46)	0.399
Uterine rupture	ND^a^		ND^a^		ND^a^		ND^a^	
**Infants**								
PTB	1.11 (0.87–1.41)	0.419	1.11 (0.91–1.37)	0.303	1.08 (0.87–1.34)	0.509	0.97 (0.77–1.23)	0.808
Very PTB	2.41 (0.55–10.51)	0.240	0.88 (0.24–3.21)	0.842	0.72 (0.22–2.31)	0.579	1.34 (0.27–6.75)	0.722
LBW	2.34 (1.37–3.98)	0.002	1.10 (0.79–1.53)	0.569	0.74 (0.54–1.02)	0.062	0.88 (0.59–1.32)	0.540
Macrosomia	ND^a^		ND^a^		ND^a^		ND^a^	
Apgar score < 7 at 1 min	3.35 (0.73–15.51)	0.122	6.45 (2.08–19.97)	0.001	0.45 (0.18–1.08)	0.074	0.27 (0.11–0.68)	0.006

*ART, assisted reproductive technology; CS, Cesarean section; VD, vaginal delivery; aRR, adjusted risk ratio; CI, confidence interval; ND, not defined; GDM, gestational diabetes mellitus; pPROM, preterm prelabor rupture of the membranes; PTB, preterm birth; LBW, low birthweight.*

*^a^Because of zero counts in one cell.*

*^b^RRs were adjusted for maternal age and body mass index at the time of delivery, interpregnancy interval, other previous intrauterine operation, and education level.*

*^c^RRs were adjusted for maternal age and body mass index at the time of delivery, interpregnancy interval, other previous intrauterine operation, education level, embryo transfer methods, and embryo developmental stage.*

*^d^RRs were adjusted for maternal age and body mass index at the time of delivery, interpregnancy interval, other previous intrauterine operation, education level, fertilization modes, and embryo developmental stage.*

*^e^RRs were adjusted for maternal age and body mass index at the time of delivery, interpregnancy interval, other previous intrauterine operation, education level, fertilization modes, and embryo transfer methods.*

### Obstetric and Perinatal Outcomes Between Different Types of Assisted Reproductive Technology Procedure Used in Cesarean Section-Assisted Reproductive Technology Group

As shown in Table 5, women with singletons conceived through ICSI were at an increased risk of velamentous placenta (aRR 2.46, 95%CI 1.35–4.48) as compared to those with singletons conceived through IVF. Women with singletons conceived through cleavage-stage ET were 1.74 times more likely to develop GDM (95%CI 1.16–2.60) than those involving singletons conceived through blastocyst-stage ET (Table 5). As shown in Table 6, no significantly increased incidence of GDM (aRR 0.54, 95%CI 0.29–1.01) was observed between twins conceived through blastocyst-stage ET and through cleavage-stage ET.

## Discussion

In this study, women with singletons in CS–ART group were at increased risk for adverse obstetric and perinatal outcomes when compared to those with singletons in CS–SC group. The risk was particularly increased for placental anomalies of implantation. In addition, the implementation of ART and previous CS interact synergistically to increase the likelihood of placenta accreta spectrum in women with singleton pregnancies. The obstetric and perinatal outcomes between different types of ART procedures used in women with previous CS were also examined: women with singletons conceived through ICSI were at increased risk of velamentous placenta compared with those with singletons conceived through IVF; whereas women with singletons conceived through cleavage-stage ET were at increased risk of GDM than those with singletons conceived through blastocyst-stage ET.

Women with singletons in CS–ART group were associated with an increased risk of maternal complications, such as gestational hypertension, GDM, velamentous placenta, placenta accreta spectrum, postpartum hemorrhage, as well as PTB, as compared with those with singletons in CS–SC group. This finding was in line with several recent cohort studies, and a 2016 meta–analysis including 50 cohort studies that showed high relative risks for adverse obstetric and perinatal outcomes in the ART group as compared with the spontaneous conception group (Qin et al., 2016; Zhu et al., 2016; Vannuccini et al., 2018; Yanaihara et al., 2018). Notably, the incidence of placenta accreta spectrum of singletons conceived through ART in our study (17.3%) was higher than that reported in a large-sample retrospective cohort study (6.9%) (Zhu et al., 2016). One of the possible explanations for this inconsistent result may be that our study was restricted to a high-risk population of women with previous CS, in contrast to previous studies. In the present study, women with singletons in the CS-ART group were 5.30 (95%CI 4.01–7.00) times more likely to develop placenta accreta spectrum than those with singletons in VD–SC group, which resulted from the positive interaction on the additive scale between the implementation of ART and previous CS. Although previous studies have identified previous CS as a risk factor for placenta accreta spectrum (Fitzpatrick et al., 2012), our study provided additional evidence suggesting that the implementation of ART and previous CS interacted synergistically to increase the likelihood of placenta accreta spectrum. This means that the joint effect of ART and previous CS exceeded the mere sum of their individual effects on placenta accreta spectrum.

The current hypothesis for the development of placenta accreta spectrum is that of a secondary defect of the endometrial–myometrial interface, leading to a failure of normal decidualization in the area of the uterine scar, allowing abnormally deep placentation (Jauniaux and Burton, 2018). Maternal pelvic factors, such as morphological, structural, and biological changes in the endometrium, are associated with infertility. Stimulation protocols or hormonal support in ART could also wholly or partly contribute to the incidence of placental disorders (Simon et al., 2003; Nakamura et al., 2015; Zhao et al., 2019; Jauniaux et al., 2020). The underlying mechanisms by which a Cesarean scar and ART interact in a synergistic manner to increase the risk of placenta accreta spectrum might require further investigation.

A recent cohort study confirmed the association between velamentous placenta and IVF and found that the odds ratio for velamentous placenta in women with IVF pregnancy was 1.72 (Yanaihara et al., 2018). However, the study involved only uncomplicated singletons conceived by IVF, and did not include ICSI. Our study established that ICSI had an enhanced effect on the incidence of velamentous placenta, as compared to IVF, in singleton pregnancies of women with previous CS. Possible mechanisms for this observation may relate to the genetic or epigenetic changes in trophectoderm cells due to ICSI, resulting in abnormal placentation (Tarín et al., 2014). Furthermore, our results indicated that cleavage-stage ET increased the risk of GDM in singleton pregnancies of women with previous CS, as compared with blastocyst-stage ET. Dysregulation of placental function may contribute to the pathogenesis of GDM (Souvannavong-Vilivong et al., 2019). Our findings raise the possibility that the improvement of uterine and embryonic synchronicity due to the prolonged *in vitro* culture of the trophectoderm cell may contribute to abnormal production or function of various placenta secrete molecules (Ming et al., 2012), which influences the pathogenesis of obstetric and perinatal outcomes. The exact mechanism by which different types of ART procedures might be related to placental abnormalities and subsequent obstetric and perinatal outcomes should be studied further.

Our study showed an increased risk for obstetric and perinatal outcomes in ART singletons as compared with spontaneously conceived neonates, but unexpectedly, we did not observe a similar trend in twin pregnancies. The reasons for this were probably as follows: (1) a lack of sufficient samples of twin pregnancies in women with previous CS; (2) most of the twins conceived naturally are monozygotic, while those conceived by ART are dizygotic. Therefore, the risk of adverse obstetric and perinatal outcomes in twin pregnancies conceived by ART compared with spontaneously conceived twins should be further studied, and conclusions should be drawn with caution.

Our findings provide valuable information for estimating and improving the safety of pregnancies in women with a Cesarean scar who seek ART, and might be useful in decision-making for women and clinical doctors to balance the risks and benefits of a Cesarean delivery in the first and subsequent births. With an enlarged sample size (> 10,000), we could adjust for confounders to enhance statistical power and thereby could provide more precise and reliable risk estimates. Our analyzed data were collected from case notes at the time of delivery, which minimized selection and recall bias. Additionally, our study indicated that ART singletons in women with previous CS carry an increased risk of adverse obstetric and perinatal outcomes, which has been poorly investigated to date.

This study had some limitations. The major weakness of this study lies in its retrospective nature and some confounders may be unavailable or unknown for adjustment. Although obstetric and perinatal outcomes during the first delivery may contribute to increased risks of adverse outcomes in the subsequent delivery, this information was only available in aggregated form in the delivery records for the second live birth. Therefore, there may have been residual confounding due to the lack of control for other potential confounding factors. It was difficult to confirm the indications for previous CS from the retrospective data. Further prospective studies are required to reduce the information bias. In addition, our database did not routinely record ultrasonographic features of Cesarean scar defect and therefore, we were unable to separately assess its role on obstetric and perinatal outcomes.

## Conclusion

In women with previous CS, clinicians should be aware of the increased risk of adverse obstetric and perinatal outcomes in pregnancies conceived by ART, particularly placental anomalies of implantation. Compared with other types of ART procedures, IVF and blastocyst-stage ET may be relatively safe for the high-risk population of women with previous CS who are undergoing ART.

## Data Availability Statement

The raw data supporting the conclusions of this article will be made available by the authors, without undue reservation.

## Author Contributions

FJ: conceptualization and methodology. YL: data curation and writing—original draft preparation. QC: writing—reviewing and editing. XH: supervision. ZW: formal analysis and software. CC and HC: investigation. All authors contributed to the article and approved the submitted version.

## Conflict of Interest

The authors declare that the research was conducted in the absence of any commercial or financial relationships that could be construed as a potential conflict of interest. The handling editor declared a shared affiliation with two of the authors YL and FJ at time of review.

## Publisher’s Note

All claims expressed in this article are solely those of the authors and do not necessarily represent those of their affiliated organizations, or those of the publisher, the editors and the reviewers. Any product that may be evaluated in this article, or claim that may be made by its manufacturer, is not guaranteed or endorsed by the publisher.
