# Supplementary material for: Obstetric and Perinatal Outcomes After Assisted Reproductive Technology in Women With Cesarean Scar
Source: Front Physiol. 2022 Feb 17;13:808079. doi: 10.3389/fphys.2022.808079 (PMC8891634; doi:10.3389/fphys.2022.808079)
Supplement: Supplementary file 1 [file Table_1.docx]

**Table S1. The effect of previous CS on obstetric and perinatal outcomes in singletons.**

|  | | **Previous CS vs Previous VD in spontaneous conception group** | | | | **Previous CS vs Previous VD in ART pregnancies group** | | | | |
| --- | --- | --- | --- | --- | --- | --- | --- | --- | --- | --- |
|  |  | **RR (95%CI)** | ***P*-value** | **aRR (95%CI)** | ***P*-value** | **RR (95%CI)** | ***P*-value** | **aRR** | **(95%CI)** | ***P*-value** |
| **Pregnancy complications** | | | | | | | | | | |
|  | **Gestational hypertension** | 1.17 (0.96-1.44) | 0.124 | 1.18 (0.96-1.44) | 0.118 | 0.81 (0.56-1.18) | 0.280 | 0.89 (0.61-1.29) | | 0.541 |
|  | **Preeclampsia** | 1.37 (1.05-1.78) | 0.020 | 1.38 (1.06-1.79) | 0.018 | 0.73 (0.39-1.35) | 0.310 | 0.77 (0.41-1.43) | | 0.409 |
|  | **GDM** | 1.31 (1.20-1.43) | <0.001 | 1.29 (1.18-1.40) | <0.001 | 1.05 (0.86-1.29) | 0.652 | 1.17 (0.96-1.42) | | 0.119 |
| **Placental anomalies of implantation** | | | | | | | | | | |
|  | **Placenta previa** | 1.75 (1.36-2.26) | <0.001 | 1.81 (1.40-2.34) | <0.001 | 1.35 (0.70-2.60) | 0.369 | 1.31 (0.68-2.53) | | 0.423 |
|  | **Low-lying placenta** | 1.09 (0.69-1.72) | 0.715 | 1.06 (0.67-1.69) | 0.824 | 0.88 (0.33-2.36) | 0.805 | 0.80 (0.30-2.16) | | 0.661 |
|  | **Velamentous placenta** | 0.71 (0.60-0.85) | <0.001 | 0.72 (0.60-0.85) | <0.001 | 0.97 (0.66-1.44) | 0.895 | 1.07 (0.72-1.58) | | 0.740 |
|  | **Placenta accreta spectrum** | 2.73 (2.31-3.21) | <0.001 | 2.70 (2.29-3.19) | <0.001 | 2.15 (1.53-3.02) | <0.001 | 2.05 (1.46-2.88) | | <0.001 |
| **Other complications** | | | | | | | | | | |
|  | **Placental abruption** | 0.67 (0.45-0.98) | 0.041 | 0.70 (0.47-1.03) | 0.072 | 0.52 (0.18-1.48) | 0.217 | 0.51 (0.18-1.46) | | 0.209 |
|  | **Postpartum hemorrhage** | 0.74 (0.42-1.31) | 0.299 | 0.75 (0.42-1.34) | 0.327 | 0.23 (0.12-0.45) | <0.001 | 0.21 (0.11-0.41) | | <0.001 |
|  | **pPROM** | 0.53 (0.48-0.58) | <0.001 | 0.52 (0.48-0.57) | <0.001 | 0.64 (0.47-0.88) | 0.005 | 0.64 (0.47-0.87) | | 0.005 |
|  | **Uterine rupture** | ND^a^ | | ND^a^ | | ND^a^ | | ND^a^ | | |
| **Infants** | | | | | | | | | | |
|  | **PTB** | 1.11 (0.97-1.26) | 0.124 | 1.05 (0.88-1.24) | 0.595 | 1.74 (1.16-2.60) | 0.007 | 1.79 (1.19-2.67) | | 0.005 |
|  | **Very PTB** | 0.95 (0.66-1.35) | 0.756 | 0.77 (0.47-1.24) | 0.282 | 1.70 (0.61-4.75) | 0.308 | 1.89 (0.66-5.39) | | 0.236 |
|  | **LBW** | 1.16 (0.95-1.40) | 0.147 | 1.01 (0.78-1.32) | 0.922 | 1.85 (1.00-3.40) | 0.049 | 1.80 (0.98-3.33) | | 0.059 |
|  | **Macrosomia** | 0.95 (0.83-1.10) | 0.485 | 0.81 (0.62-1.07) | 0.136 | 0.63 (0.41-0.98) | 0.041 | 0.63 (0.41-0.98) | | 0.042 |
|  | **Apgar score <7 at 1 minute** | 1.24 (0.89-1.73) | 0.198 | 1.03 (0.63-1.69) | 0.904 | 0.76 (0.27-2.11) | 0.596 | 0.78 (0.27-2.24) | | 0.647 |

ART, assisted reproductive technology; CS, Cesarean section; VD, vaginal delivery; aRR, adjusted risk ratio; CI, confidence interval; ND, not defined; GDM, gestational diabetes mellitus; pPROM, preterm prelabor rupture of the membranes; PTB, preterm birth; LBW, low birthweight.

For comparison between spontaneous conception group, RRs were adjusted for maternal age and body mass index at the time of delivery, interpregnancy interval, other previous intrauterine operation, and education level.

For comparison between ART pregnancies group, RRs were adjusted for maternal age and body mass index at the time of delivery, interpregnancy interval, other previous intrauterine operation, education level, fertilization modes, embryo transfer methods and embryo developmental stage.

^a^ Because of zero counts in one cell.
