# Supplementary material for: Obstetric and Perinatal Outcomes After Assisted Reproductive Technology in Women With Cesarean Scar
Source: Front Physiol. 2022 Feb 17;13:808079. doi: 10.3389/fphys.2022.808079 (PMC8891634; doi:10.3389/fphys.2022.808079)
Supplement: Supplementary file 3 [file Table_3.docx]

**Table S3. Interaction between ART procedures and previous CS on risk of obstetric and perinatal outcomes in subsequent twin pregnancies.**

|  | | | **Twin pregnancies** | | | | |
| --- | --- | --- | --- | --- | --- | --- | --- |
|  |  |  | **Previous VD** | | **Previous CS** | | **RERI (95% CI)** |
|  |  |  | **No. with/**  **without Outcome** | **aRR (95% CI)** | **No. with/**  **without Outcome** | **aRR (95% CI)** |  |
|  | **Gestational hypertension** | **Spontaneous conception** | 8/87 | 1.0 | 10/60 | 1.78 (0.74-4.29) | ND^a^ |
|  |  | **ART** | 49/215 | 1.78 (0.74-4.30) | 32/142 | 1.96 (0.84-4.60) |  |
|  | **Preeclampsia** | **Spontaneous conception** | 4/91 | 1.0 | 7/63 | 2.24 (0.66-7.57) | ND^a^ |
|  |  | **ART** | 35/229 | 2.80 (0.86-9.12) | 14/160 | 1.52 (0.48-4.87) |  |
|  | **GDM** | **Spontaneous conception** | 17/78 | 1.0 | 14/56 | 0.96 (0.50-1.83) | ND^a^ |
|  |  | **ART** | 77/187 | 2.06 (1.20-3.52) ^b^ | 48/126 | 1.57 (0.82-3.00) |  |
|  | **Placenta previa** | **Spontaneous conception** | 1/94 | 1.0 | 0/70 | ND^a^ | ND^a^ |
|  |  | **ART** | 8/256 | 2.15 (0.20-22.86) | 16/158 | 4.64 (0.59-36.23) |  |
|  | **Low-lying placenta** | **Spontaneous conception** | 1/94 | 1.0 | 0/70 | ND^a^ | ND^a^ |
|  |  | **ART** | 4/260 | 0.76 (0.04-14.18) | 5/169 | 2.63 (0.27-25.47) |  |
|  | **Velamentous placenta** | **Spontaneous conception** | 5/90 | 1.0 | 6/64 | 1.57 (0.49-5.03) | ND^a^ |
|  |  | **ART** | 13/251 | 0.39 (0.13-1.22) | 23/151 | 2.47 (0.91-6.70) |  |
|  | **Placenta accreta spectrum** | **Spontaneous conception** | 12/83 | 1.0 | 9/61 | 1.02 (0.45-2.28) | ND^a^ |
|  |  | **ART** | 31/233 | 0.85 (0.37-1.94) | 22/152 | 1.77 (0.81-3.89) |  |
|  | **Placental abruption** | **Spontaneous conception** | 0/95 | 1.0 | 1/69 | ND^a^ | ND^a^ |
|  |  | **ART** | 3/261 | ND^a^ | 1/173 | ND^a^ |  |
|  | **Postpartum hemorrhage** | **Spontaneous conception** | 0/95 | 1.0 | 0/70 | ND^a^ | ND^a^ |
|  |  | **ART** | 13/251 | ND^a^ | 10/164 | ND^a^ |  |
|  | **pPROM** | **Spontaneous conception** | 16/79 | 1.0 | 7/63 | 0.57 (0.24-1.35) | ND^a^ |
|  |  | **ART** | 44/220 | 0.61 (0.27-1.37) | 36/138 | 1.35 (0.70-2.61) |  |
|  | **Uterine rupture** | **Spontaneous conception** | 0/95 | ND^a^ | 1/69 | ND^a^ | ND^a^ |
|  |  | **ART** | 0/264 | ND^a^ | 0/174 | ND^a^ |  |
|  | **PTB** | **Spontaneous conception** | 98/92 | 1.0 | 82/58 | 1.19 (0.91-1.57) | ND^a^ |
|  |  | **ART** | 296/232 | 1.11 (0.84-1.47) | 240/108 | 1.24 (0.98-1.58) |  |
|  | **Very PTB** | **Spontaneous conception** | 4/186 | 1.0 | 4/136 | 1.36 (0.20-9.40) | ND^a^ |
|  |  | **ART** | 18/510 | 1.62 (0.36-7.36) | 24/324 | 4.04 (0.93-17.63) |  |
|  | **LBW** | **Spontaneous conception** | 28/162 | 1.0 | 27/113 | 1.31 (0.68-2.52) | ND^a^ |
|  |  | **ART** | 185/343 | 2.26 (1.35-3.80) ^b^ | 149/199 | 3.53 (2.11-5.92) ^b^ |  |
|  | **Macrosomia** | **Spontaneous conception** | 0/190 | 1.0 | 0/140 | ND^a^ | ND^a^ |
|  |  | **ART** | 0/528 | ND^a^ | 0/348 | ND^a^ |  |
|  | **Apgar score <7 at 1 minute** | **Spontaneous conception** | 8/182 | 1.0 | 3/137 | 0.51 (0.10-2.68) | ND^a^ |
|  |  | **ART** | 20/508 | 0.90 (0.34-2.41) | 25/323 | 1.76 (0.60-5.12) |  |

ART, assisted reproductive technology; CS, Cesarean section; VD, vaginal delivery; aRR, adjusted risk ratio; CI, confidence interval; No., number; RERI, relative excess risk due to interaction; ND, not defined; GDM, gestational diabetes mellitus; pPROM, preterm prelabor rupture of the membranes; PTB, preterm birth; LBW, low birthweight.

For comparison between spontaneous conception group, RRs were adjusted for maternal age and body mass index at the time of delivery, interpregnancy interval, other previous intrauterine operation, and education level.

For comparison between ART pregnancies group, RRs were adjusted for maternal age and body mass index at the time of delivery, interpregnancy interval, other previous intrauterine operation, education level, fertilization modes, embryo transfer methods and embryo developmental stage.

^a^ Because of zero counts in one cell.

^b^ Statistically significant (*P* < 0.05).
